# Phenotype and Genotype Correlations in Inherited Retinal Diseases: Population-Guided Variant Interpretation, Variable Expressivity and Incomplete Penetrance

**DOI:** 10.3390/genes11111274

**Published:** 2020-10-29

**Authors:** Jamie M. Ellingford, Robert B. Hufnagel, Gavin Arno

**Affiliations:** 1 North West Genomic Laboratory Hub, Manchester Centre for Genomic Medicine, Manchester University NHS Foundation Trust, St Mary’s Hospital, Manchester M13 9WL, UK; 2 Division of Evolution and Genomic Sciences, Neuroscience and Mental Health Domain, School of Biological Sciences, Faculty of Biology, Medicine and Health, University of Manchester, Manchester M13 9PT, UK; 3 Ophthalmic Genetics and Visual Function Branch, National Eye Institute, National Institutes of Health, Bethesda, MD 20016, USA; 4 University College London Institute of Ophthalmology, London EC1V 9EL, UK; 5 Moorfields Eye Hospital, London EC1V 2PD, UK

Inherited retinal diseases (IRDs) are a diverse and variable group of rare human disorders. The common theme between IRDs is that individuals develop visual impairment as a result of dysfunction or degeneration of the retina, a highly specialised tissue at the back of the eye which enables vision through the conversion of light energy into neuronal signals. However, the spectrum of disorders included within the IRD umbrella is extremely diverse, ranging from severe and early onset blindness (e.g., Leber Congenital Amaurosis, MIM #204000) through to late onset disorders which have noticeable but mild changes to visual acuity or visual field (e.g., late onset macular dystrophy, MIM #153700). IRDs can also be a part of multi-systemic disorders, including disorders which involve hearing loss (MIM #276901), renal failure (MIM #609254) and malfunction in development (MIM #209900). The IRDs are extremely genetically heterogeneous, with over 270 genes characterised as a cause of disease when disrupted by pathogenic genetic variation (RetNet, https://sph.uth.edu/retnet/).

A large portion of the variability in phenotypic presentation associated with IRDs can be attributed to the specific gene carrying the disease-causing variation. For example, individuals with pathogenic dominant variants in *BEST1* are expected to have central visual acuity impairment as a result of the build-up of fatty yellow deposits in the retina (lipofuscin), which results in a stereotypical appearances of the retina when visualised through microscopic and photographic techniques [1], whereas pathogenic variants in *IQCB1* are expected to cause congenital or early childhood blindness followed by end-stage renal failure during adolescence of early adulthood [2,3]. This is largely due to the specific function and expression of these genes in various tissues throughout the body. For some genes associated with diverse phenotypes, e.g., *ABCA4* and *USH2A*, allelic hierarchy models exist, whereby specific variants and combinations of variants can be classified as ‘mild’, ‘moderate’, ‘severe’ (*ABCA4* [4]), or ‘syndromic’ versus ‘non-syndromic’ (*USH2A* [5,6]). Understanding the nature and the intricacies of these phenotypic and genotypic correlations in individuals with IRDs is vitally important for the management and counselling of patients. Such understanding can help individuals prepare for how their disease will progress and to understand at an early age if extra-ocular complications may be a concern in later life.

A major theme of the papers presented in this guest issue of *Genes* is to characterise some of the ethnic and genetic factors which impact *variable expressivity* in gene-phenotype associations. The authors of these articles make significant efforts to characterise phenotypic differences and similarities between individuals carrying pathogenic genetic variants in the same IRD genes. Examples of the interesting conclusions drawn from these analyses include the variability in phenotypes associated with the same genetic variant, and descriptions of the most prevalent disease-causing variants in certain ethnic groups. For example, Motta et al. [7] describe clinical findings in Brazilian patients with LCA caused by the *RPE65* missense variants, c.247T > C (p.Phe83Leu) and c.560G > A (p.Gly187Glu). They show that the increased frequency of these *RPE65* missense variants in Brazilian patients with early onset blindness clarifies variant interpretation in that the apparent enrichment of these alleles in the disease population can alter the classification from ‘variants of uncertain significance’ to ‘likely pathogenic’. Moreover, Ur Rehman et al. [8] describe how the genetic architectures of IRDs can differ between distinct regions of Pakistan. The authors attribute the presence of two founder mutations (*ABCA4* p.Gly72Arg and *NMNAT1* p.Val9Met) in north-west Pakistan due to different ancestral histories between different parts of the country, which remain largely isolated today due to cultural, linguistic and geographical reasons. Both studies further reinforce the need for increasingly diverse reference genome datasets to more accurately identify and interpret genomic variants in the context of rare disease. Other studies in this issue describe genotypic findings from specific ancestral cohorts, including a large genotypic study from Irish patients [9], descriptions of macular genetic disease associations in defined populations [10,11], and the description of detailed phenotypic presentations associated with specific genetic variants [12,13].

Interestingly, two of the studies in this issue describe findings that the same genetic variant can lead to extreme variability in phenotypic presentation. Zupan et al. [14] focus on the diverse clinical presentation associated with a nonsense variant in *USH2A* (p.Trp3955Ter), which is the most frequent cause of *USH2A*-related Usher syndrome in Slovenia. The authors construct four distinct haplotypes associated with p.Trp3955Ter homozygous variants in Slovenian patients and describe a range of clinical findings in these patients. This includes a range of ages at which visual impairments were first diagnosed (4–42 years) and a broad range of disease manifestations in later life, ranging from good central vision at the age of 62 to severe blindness at this age. Green et al. [15] focus on variants which show *incomplete penetrance.* Through the comparison of disease and ‘healthy’ population genomic datasets, the authors show that in some cases the same genetic variant may result in disease, whereas in other individuals it causes no obvious signs of eye disease. The authors go on to demonstrate that variants in IRD genes may be modulated by the impact of other factors that alter the expression levels of these IRD genes. For example, their analyses show that 125 unique genomic variants are described in a disease database for *PRPF31*, a gene known as a cause of autosomal dominant retinitis pigmentosa [16]. They identified that a considerable number of *PRPF31* pathogenic variants also exist in ‘healthy’ populations, and demonstrate that the level of variability in expression profiles of *PRPF31* in retinal tissue is higher (72.6 local coefficient of variation, *LCV*) than is detected on average for genes that do not show significant levels of incomplete penetrance (67.8 LCV). It is well described that the severity of disease in *PRPF31* is correlated with the level of expression of the wild-type allele, and that other factors, such as non-coding regulatory region variants, may influence the expression levels of *PRPF31* [17].

The concepts of *variable expressivity* and *incomplete penetrance* are fascinating and important revelations in hereditary eye diseases. Whilst some well-known examples exist, including *PRPF31*, unpicking the additional factors which impact how and whether disease manifests in the presence of specific genetic variants is likely to be a fast moving area of study over the coming years. Incorporating factors such as the percentage splice inclusion of exons that carry pathogenic variants, particularly for genes showing biases towards tissue-specific isoforms, e.g., *DYNC2H1* [18], may help elucidate the variable expressivity and the tissue-specific presentation of some genetic variants. Furthering understanding in this area may begin to pave the way for novel diagnostic, treatment and patient management approaches.

## References

[1] Petrukhin K., Koisti M.J., Bakall B., Li W., Xie G., Marknell T., Sandgren O., Forsman K., Holmgren G., Andreasson S. (1998). Identification of the gene responsible for Best macular dystrophy. Nat. Genet..

[2] Otto E.A., Loeys B., Khanna H., Hellemans J., Sudbrak R., Fan S., Muerb U., O’Toole J.F., Helou J., Attanasio M. (2005). Nephrocystin-5, a ciliary IQ domain protein, is mutated in Senior-Loken syndrome and interacts with RPGR and calmodulin. Nat. Genet..

[3] Ellingford J.M., Sergouniotis P.I., Lennon R., Bhaskar S., Williams S.G., Hillman K.A., O’Sullivan J., Hall G., Ramsden S.C., Lloyd I.C. (2015). Pinpointing clinical diagnosis through whole exome sequencing to direct patient care: A case of Senior-Loken syndrome. Lancet.

[4] Cornelis S.S., Bax N.M., Zernant J., Allikmets R., Fritsche L.G., den Dunnen J.T., Ajmal M., Hoyng C.B., Cremers F.P. (2017). In Silico Functional Meta-Analysis of 5,962 ABCA4 Variants in 3,928 Retinal Dystrophy Cases. Hum. Mutat..

[5] Lenassi E., Vincent A., Li Z., Saihan Z., Coffey A.J., Steele-Stallard H.B., Moore A.T., Steel K.P., Luxon L.M., Heon E. (2015). A detailed clinical and molecular survey of subjects with nonsyndromic USH2A retinopathy reveals an allelic hierarchy of disease-causing variants. Eur. J. Hum. Genet..

[6] Molina-Ramírez L.P., Lenassi E., Ellingford J.M., Sergouniotis P.I., Ramsden S.C., Bruce I.A., Black G.C.M. (2020). Establishing Genotype-phenotype Correlation in USH2A-related Disorders to Personalize Audiological Surveillance and Rehabilitation. Otol. Neurotol..

[7] Motta F.L., Martin R.P., Porto F.B.O., Wohler E.S., Resende R.G., Gomes C.P., Pesquero J.B., Sallum J.M.F. (2020). Pathogenicity Reclasssification of RPE65 Missense Variants Related to Leber Congenital Amaurosis and Early-Onset Retinal Dystrophy. Genes.

[8] Rehman A.U., Peter V.G., Quinodoz M., Rashid A., Khan S.A., Superti-Furga A., Rivolta C. (2020). Exploring the Genetic Landscape of Retinal Diseases in North-Western Pakistan Reveals a High Degree of Autozygosity and a Prevalent Founder Mutation in ABCA4. Genes.

[9] Whelan L., Dockery A., Wynne N., Zhu J., Stephenson K., Silvestri G., Turner J., O’Byrne J.J., Carrigan M., Humphries P. (2020). Findings from a Genotyping Study of Over 1000 People with Inherited Retinal Disorders in Ireland. Genes.

[10] Tracewska A.M., Kocyła-Karczmarewicz B., Rafalska A., Murawska J., Jakubaszko-Jablonska J., Rydzanicz M., Stawiński P., Ciara E., Khan M.I., Henkes A. (2019). Genetic Spectrum of ABCA4-Associated Retinal Degeneration in Poland. Genes.

[11] Shoshany N., Weiner C., Safir M., Einan-Lifshitz A., Pokroy R., Kol A., Modai S., Shomron N., Pras E. (2019). Rare Genetic Variants in Jewish Patients Suffering from Age-Related Macular Degeneration. Genes.

[12] Fakin A., Šuštar M., Brecelj J., Bonnet C., Petit C., Zupan A., Glavač D., Jarc-Vidmar M., Battelino S., Hawlina M. (2019). Double Hyperautofluorescent Rings in Patients with USH2A-Retinopathy. Genes.

[13] Habibi I., Falfoul Y., Todorova M.G., Wyrsch S., Vaclavik V., Helfenstein M., Turki A., Matri K.E., Matri L.E., Schorderet D.F. (2019). Clinical and Genetic Findings of Autosomal Recessive Bestrophinopathy (ARB). Genes.

[14] Zupan A., Fakin A., Battelino S., Jarc-Vidmar M., Hawlina M., Bonnet C., Petit C., Glavač D. (2019). Clinical and Haplotypic Variability of Slovenian. Genes.

[15] Green D.J., Sallah S.R., Ellingford J.M., Lovell S.C., Sergouniotis P.I. (2020). Variability in Gene Expression is Associated with Incomplete Penetrance in Inherited Eye Disorders. Genes.

[16] Vithana E.N., Abu-Safieh L., Allen M.J., Carey A., Papaioannou M., Chakarova C., Al-Maghtheh M., Ebenezer N.D., Willis C., Moore A.T. (2001). A human homolog of yeast pre-mRNA splicing gene, PRP31, underlies autosomal dominant retinitis pigmentosa on chromosome 19q13.4 (RP11). Mol. Cell.

[17] Wheway G., Douglas A., Baralle D., Guillot E. (2020). Mutation spectrum of PRPF31, genotype-phenotype correlation in retinitis pigmentosa, and opportunities for therapy. Exp. Eye. Res..

[18] Vig A., Poulter J.A., Ottaviani D., Tavares E., Toropova K., Tracewska A.M., Mollica A., Kang J., Kehelwathugoda O., Paton T. (2020). DYNC2H1 hypomorphic or retina-predominant variants cause nonsyndromic retinal degeneration. Genet. Med..

